# Seasonal Variations in Water-Quality, Antibiotic Residues, Resistant Bacteria and Antibiotic Resistance Genes of *Escherichia coli* Isolates from Water and Sediments of the Kshipra River in Central India

**DOI:** 10.3390/ijerph15061281

**Published:** 2018-06-17

**Authors:** Vishal Diwan, Nada Hanna, Manju Purohit, Salesh Chandran, Emilia Riggi, Vivek Parashar, Ashok J. Tamhankar, Cecilia Stålsby Lundborg

**Affiliations:** 1 Department of Public Health and Environment, R.D. Gardi Medical College, Ujjain 456006, India; vivek.p@rdgmc.edu.in; 2 Department of Public Health Sciences, Global Health, Health Systems and Policy (HSP): Medicines Focusing Antibiotics, Karolinska Institutet, Stockholm 171 77, Sweden; nada.hanna@ki.se (N.H.); manju.purohit@ki.se (M.P.); ejetee@gmail.com (A.J.T.); Cecilia.Stalsby.Lundborg@ki.se (C.S.L.); 3 International Centre for Health Research, Ujjain Charitable Trust Hospital and Research Centre, Ujjain 456001, India; 4 Department of Pathology, R.D. Gardi Medical College, Ujjain 456006, India; 5 HLL Biotech Ltd., Integrated Vaccines Complex, Melaripakkam (Post), Thirukalukundram Taluk, Chengalpattu 603001, India; saleshp@gmail.com; 6 Department of Brain and Behavioural Sciences, University of Pavia, Pavia 27100, Italy; emilia.riggi01@ateneopv.it; 7 Research Center in Epidemiology and Preventive Medicine (EPIMED), University of Insubria, Varese 21100, Italy; 8 Indian Initiative for Management of Antibiotic Resistance, Department of Environmental Medicine, R.D. Gardi Medical College, Ujjain 456006, India

**Keywords:** antibiotic residues, antibiotic resistance, antibiotic resistance genes, seasonal variation, Kshipra River, India

## Abstract

Objectives: To characterize the seasonal variation, over one year, in water-quality, antibiotic residue levels, antibiotic resistance genes and antibiotic resistance in *Escherichia coli* isolates from water and sediment of the Kshipra River in Central India. Methods: Water and sediment samples were collected from seven selected points from the Kshipra River in the Indian city of Ujjain in the summer, rainy season, autumn and winter seasons in 2014. Water quality parameters (physical, chemical and microbiological) were analyzed using standard methods. High-performance liquid chromatography–tandem mass spectrometry was used to determine the concentrations of antibiotic residues. In river water and sediment samples, antibiotic resistance and multidrug resistance patterns of isolated *E. coli* to 17 antibiotics were tested and genes coding for resistance and phylogenetic groups were detected using multiplex polymerase chain reaction. One-way analysis of variance (ANOVA) and Fisher tests were applied to determine seasonal variation. Results: In river water, seasonal variation was significantly associated with various water quality parameters, presence of sulfamethoxazole residues, bacteria resistant to ampicillin, cefepime, meropenem, amikacin, gentamicin, tigecycline, multidrug resistance and CTX-M-1 gene. The majority of the Extended Spectrum Beta-Lactamase (ESBL)-producing *E. coli* isolates from river water and sediment in all different seasons belonged to phylogenetic group A or B1. Conclusions: Antibiotic pollution, resistance and resistance genes in the Kshipra River showed significant seasonal variation. Guidelines and regulatory standards are needed to control environmental dissemination of these “pollutants” in this holy river.

## 1. Introduction

The occurrence of antibiotics (in this paper we use the term antibiotics in its wider version that includes substances with antibacterial properties that may be produced by microorganisms or may be synthetically or semi-synthetically produced compounds) in the aquatic environment has been considered as one of the emerging issues in environmental pollution [1]. After use, antibiotics are partially metabolized and subsequently excreted by humans and animals and they enter the aquatic environment as unchanged compounds as well as a mixture of their metabolites [2]. In general, 50–80% of total parent compounds are excreted as a mixture of metabolite-conjugated compounds [3]. Antibiotics are released into the environment through different routes, such as sewage effluent, agricultural activities, surface run-off, and waste discharge of animal facilities [4,5,6,7]. Specifically, these pollutants can flow into the river either through the discharge of municipal and community sewage or through manufacturing wastewater effluents [8]. Antibiotic residues have been detected in many environmental niches, including river water and aquatic sediments [9,10]. The occurrence and the fate of antibiotic residues in aquatic systems is affected by the antibiotics’ physicochemical properties, partition characteristics and environmental behavior [11]. Further, various factors can affect the temporal variation in antibiotic concentration such as half-life, metabolism, excretion, temperature and flow dynamics [12,13].

Antibiotic residues may lead to adverse effects in the environment, such as the development of resistant bacteria/resistance genes [14] and may eventually become a threat to human health. These resistant bacteria might be transmitted from environment to humans and animals and vice versa [15]. *Escherichia coli (E. coli)* forms part of the bacterial commensal flora of the human and animal gut, as well as exists in the environment. It acts as the predominant reservoir of antibiotic resistance genes and are easily transferable to pathogenic bacteria [16]. Previous studies have reported that antibiotic resistant bacteria/resistance genes have been found in the aquatic environment worldwide [17,18,19]. Importantly, studies have shown that the selection and transfer of resistant bacteria are not favored only at high antibiotic concentrations [20]; the exposures to very low levels of antibiotic concentrations below the minimal inhibitory concentration (MIC) in various environmental compartments are sufficient to maintain antibiotic resistant bacteria [21].

In many parts of the world, rivers play important roles in a country’s economic growth and are useful in number of ways. In many settings, rivers are considered sacred and are used for religious festivals including mass-gatherings [22]. However, they can also act as a disposal site for municipal and manufacturing waste. In India, very high antibiotic pollutant residue levels have been reported in rivers such as tributaries of the Manjra River, the Tamariparani River [23,24,25] and the Musi River [26]. Previous studies showed that Kshipra River water is of medium to bad quality which leads to significant environmental and health risk to the rural communities [27,28]. The Kshipra River hosts many religious festivals including Simhastha Mahakumbh Mela, which attracts millions of pilgrims from all around the world to bathe in this holy river and is organized every twelve years. This gives rise to massive mass bathes further deteriorating water quality [29,30].

The objective of present study was to characterize the seasonal variation, over one year, of water-quality, antibiotic residue levels, antibiotic resistance, and antibiotic resistance genes of *E. coli* isolates from water and sediment in the Kshipra River in Central India.

## 2. Material and Methods

The methods are described in brief here. Detailed description of the methods is available in the protocol paper published in this context [31].

### 2.1. Setting

The study was conducted at the city of Ujjain, Madhya Pradesh, India located on the banks of the Kshipra River, by sampling the water and sediment of the river over a period of one year in 2014. Kshipra is a river with which religious sentiments are attached. Therefore, several mass-gatherings are organized throughout the year on the banks of the Kshipra River at Ujjain and thousands of pilgrims take a holy dip all the year round.

### 2.2. Sample Collection

**Figure 1:**
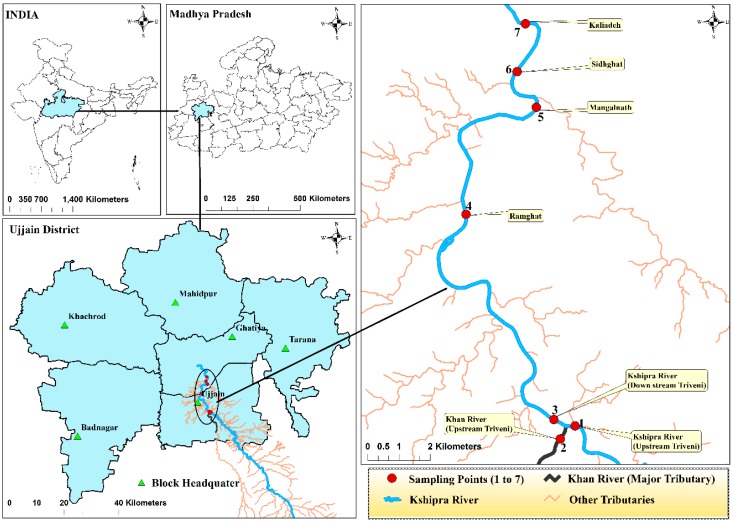
Geographical location of the study sites. The map shows (clockwise) India, Madhya Pradesh, Ujjain District and the sampling points for the Kshipra River, respectively.

Water samples from the river were collected in duplicates from seven selected points (Figure 1). Sample collection procedures are described in detail in our previously published river protocol paper [31]. Sediment samples were also collected from all seven points using Ekman Dredge sediment sampler. Sampling was done once each during the four seasons of the year 2014; summer (29 May 2014), rain (15 July 2014), autumn (10 October 2014) and winter (22 December 2014). The numbers of samples collected from water and sediment in each season are presented in Table 1 and Table 2.

### 2.3. Sample Analysis

#### 2.3.1. Water Quality Parameters Measured in the Field

The following water quality parameters were tested immediately after the collection of samples: pH, total dissolved solids, conductivity, free carbon dioxide, carbonate alkalinity, ambient and water temperature and dissolved oxygen .The method of measurement of above parameters is described elsewhere [31]. 

#### 2.3.2. Water and Sediment Quality Parameters Examined in the Laboratory

The analysis of remaining water parameters such as, hardness, chloride, turbidity, nitrate nitrogen alkalinity, nitrate nitrogen, available phosphorous, chemical oxygen demand (COD), total suspended solids (TSS), biochemical oxygen demand (BOD), and total phosphorous was done at the Central Research Laboratory of R.D. Gardi Medical College, Ujjain. The method of measurement of above parameters is described in our previously published protocol paper [31].

#### 2.3.3. Antibiotic Residue Analysis

River water and sediment samples were analyzed for six antibiotics, namely ceftriaxone, ofloxacin, ciprofloxacin, norfloxacin, sulfamethoxazole and metronidazole. These antibiotics were selected based on antibiotic residues found in the same geographical area [32,33], environmental stability, the known and suspected environmental impact of an antibiotic and the degree of antibiotic metabolism [34]. The detailed method of antibiotic residues analysis is mentioned elsewhere [31,32,33]. 

In brief, every water sample was homogenized by mixing thoroughly. 50 mL of the homogenized sample was filtered through 0.45 µm membrane filter paper with the help of filtration assembly. Sample was acidified with 1 N H_2_SO_4_ to pH 3. Sample was loaded on activated C-18 cartridge. (activated with 5 mL methanol, 5 mL methanol/water (50:50) followed by 5 mL acidified water at pH 3). Cartridge was washed with 5 mL of acidified water and adsorbed compounds were eluted with 5 mL of 5% Triethylamine in methanol. The elutant was evaporated to dryness with a gentle stream of Nitrogen gas at 50 °C. The residue was reconstituted with Acetonitrile to make final volume of 1 mL. 

Sediment samples were homogenized by mixing thoroughly. 100 mL of acidified water (pH 3.5 with phosphoric acid) was added to 20 g of samples shaken for 1 h. and filtered through Whatman filter paper no. 41 with the help of filtration assembly. A Sample was loaded on activated C-18 cartridge. (Cartridge activation was done bypassing 5 mL 1:1 methanol: water followed by 5 mL of acidified water of pH 3.0). Cartridge was washed with 5 mL of acidified water and adsorbed compounds were eluted with 5 mL of 5% Triethylamine in methanol. The elutant was evaporated to dryness with a gentle stream of Nitrogen gas at 50 °C. The residue was reconstituted with Acetonitrile to make final volume of 2 mL. 

Antibiotic residues were detected by using solid-phase extraction followed by liquid chromatography tandem-mass spectrometry (LC-MS/MS) (Waters 2695 Series Alliance Quaternary Liquid Chromatography System, Waters, Milford, MA, USA) with a triple quadruple mass spectrometer (Quatro-micro API, Micromass, Manchester, UK) equipped with an electro-spray interface and Masslynx 4.1 software (Micromass, Manchester, UK) for data acquisition and processing.

The Limits of quantification (LOQ in µg/L) and limits of detection (LOD µg/L) for antibiotics tested in water samples were- metronidazole—0.05 and 0.01, sulfamethoxazole—0.08 and 0.01, norfloxacin—0.1 and 0.01, ciprofloxacin—0.1 and 0.01, ofloxacin—0.1 and 0.01, ceftriaxone—20 and 1 respectively. The Limits of quantification (LOQ µg/kg) and limits of detection (LOD ug/kg) for antibiotics tested in sediment samples were—metronidazole—0.05 and 0.05, sulfamethoxazole—0.08 and 0.05, norfloxacin—0.1 and 0.05, ciprofloxacin—0.1 and 0.05, ofloxacin—0.1 and 0.05, ceftriaxone—20 and 0.25 respectively. 

#### 2.3.4. Microbiological Methods

The isolation and identification of *E. coli* was performed according to standard methods [35]. The enumeration of total coliform and total *E. coli* count in colony-forming units (CFUs) per 100 mL was estimated according to previously published methods. Resistance to ampicillin, cefotaxime, ceftazidime, Cefepime, nalidixic acid, ciprofloxacin, nitrofurantoin, gentamicin, amikacin, tetracycline, tigecycline, imipenem, meropenem, co-trimoxazole, sulfamethiazole, ceftazidime/clavulanic acid, cefotaxime/clavulanic acid, colistin, ceftriaxone was conducted by Kirby Bauer disc-diffusion method. Clinical and Laboratory Standard Institute guidelines was used to measure and interpreted zone diameter of bacterial growth inhibition [36].

#### 2.3.5. Molecular Methods

##### DNA Extraction

The isolation and subsequent DNA extraction of six *E. coli* isolates per surface water and sediment sample was carried out for the detection of antibiotic resistance coding genes. A single colony of *E. coli* was isolated from antibiotic resistant strains and stored at −80 °C. DNA extraction was carried out using the heat lysis method. DNA was extracted from *E. coli* isolates by using the boiling method. Briefly, a few colonies of each bacterial strain was suspended in 200 μL of distilled water and heated at 100 °C for 10 min followed by centrifugation at 14,000 rpm for 10 min and the recovered supernatant was frozen at −20 °C until use.

##### PCR Detection of Genes

*E. coli* phenotypically identified as ESBL producer was tested to detect the presence of ESBL-coding genes *bla*CTX-M, *bla*SHV and *bla*TEM and plasmid mediated quinolone resistance genes *qnr*A, *qnr*B, and *qnr*S, were detected as described by Chandran et al. (2014) [37]. Detection of sulfonamide resistance genes (*sul I* and *sul II*), Carabapenemase (VIM and NDM) was performed by using simplex PCR assays. The primer sequences, PCR conditions and amplicon sizes are mentioned in earlier publications [37,38]. Phylogenetic grouping of cephalosporin- and quinolone resistant *E. coli* isolates was carried out using multiplex PCR based on *chuA*, *yjaA* and *TspE4C2* genes [39]. The reaction mixture for PCR amplification contained 12.5 μL of PCR Master Mix (Taq PCR master mix kit, Qiagen, Hilde, Germany), 0.5 μL each of oligonucleotide primer (Integrated DNA Technologies, Coralville, IA, USA), 5 μL of template DNA and 6.5 μL of nuclease free water to constitute a total reaction volume of 25 μL. All PCR assays were performed on a thermocycler (Eppendorf mastercycler gradient, Hamburg, Germany) and each run included a negative control. Amplicons of each sample (5 μL) were mixed with 2 μL loading dye and resolved on 1.8% agarose gel containing 5 μL SYBR Safe DNA Gel Stain (Thermo Fisher Scientific Inc., Waltham, MA, USA). A 100-bp marker (Thermo Scientific, Waltham, MA, USA) was also included for DNA band size estimation. All gels were run in 0.5× TAE buffer at 90 V for 45 min and visualized by Gel documentation system (Gel Doc System, Bio-Rad Laboratories, Inc., Hercules, CA, USA). 

### 2.4. Data Management and Statistical Analysis

Descriptive statistics was used to present data as mean and its range for continuous data, while listed categorical variables are presented as numbers and percentages to determine seasonal variation. To determine seasonal variation, analysis of variance (ANOVA) for continuous variables and Fisher test for categorical variables were applied. The results are presented in the tables with the corresponding *p*-value and significant association determined by *p*-values < 0.05. For sulfamethoxazole, the seasonal variation was also adjusted for some water quality parameters.

## 3. Results

### 3.1. Water Quality

Significant (*p* < 0.05) seasonal variation in physico-chemical parameters such as pH, air temperature, water temperature, conductivity, total dissolve solids, turbidity, total alkalinity, chloride, total hardness, calcium hardness, magnesium hardness, nitrate nitrogen, total phosphorus, ortho phosphorus, organic phosphorus and bacteriological parameters such as total coliform, and total *E. coli* was observed for Kshipra River water during the one year study (Table S1). 

### 3.2. Antibiotic Residues

A total of four antibiotics were detected in the river water and sediment and there was seasonal variation in the presence of antibiotic residues in the river water and river sediment. In the river water, sulfamethoxazole was detected at higher levels in autumn (2.75 µg/L) and winter (2.18 µg/L) compared to that in summer (1.39 µg/L) and the rainy season (0.04 µg/L). The concentrations of sulfamethoxazole were significantly different between the different seasons (*p* < 0.05) and this seasonal difference had an associative relationship with pH (*p* < 0.05). Norfloxacin and ofloxacin were found only in autumn at levels of 0.66 µg/L, 0.99 µg/L, respectively. In addition, 50% of samples showed residual β-lactams (>5 ppb) in summer, (Table 1 and Table 2). In sediment, 1.39 (0–9.74) µg/Kg of ofloxacin and 1.18 (0–8.23) µg/Kg of sulfamethoxazole were detected in winter, and 50% of samples contained residual β-lactam (>5 ppb) in rainy season.

### 3.3. Antibiotic Resistance

There was a significant (*p* < 0.05) seasonal variation in resistance to ampicillin, cefepime, meropenem, amikacin, gentamicin, tigecycline and in multidrug resistance (*p* < 0.05) as shown in Table 3. Multi drug resistance (MDR) was higher in autumn compared to other seasons. For cefotaxime, imipenem, meropenem, nalidixic acid, gentamicin, nitrofurantoin, and tigecycline resistance was higher in winter than in other seasons. Of the total isolates, 12%were extended spectrum beta-lactamase (ESBL) producers, and 24% were MDR.

In sediment, the percentage of resistance to ampicillin (33%), ciprofloxacin (25%), nalidixic acid (40%), tetracycline (29%), sulfamethizole (25%), and co-trimoxazole (29%) as well as MDR (34%) was higher in autumn compared to other seasons. The percentage of resistance to cefotaxime (46%), imipenem (5%), meropenem (28%), amikacin (5%), gentamicin (5%), and tigecycline (5%) was higher in winter than in other seasons. Meropenem resistance was significantly different (*p* < 0.05) among various seasons (Table 4).

### 3.4. Antibiotic Resistance Genes

There was no significant difference in the occurrence of the various studied genes in bacterial isolates sampled during the various seasons from river water. Occurrence of ESBL-coding gene *bla*CTX-M-1 was recorded in all seasons. The other ESBL-coding genes *bla*CTX-M-2, *bla*CTX-M-9 were not detected in any of the samples in any of the seasons. Among plasmid-mediated quinolone-resistance genes *qnr S* was found in all seasons, while *qnr B* was only found in summer. Sulfonamide resistance coding genes, *sul1* and *sul2*, were found in river water in all seasons, while carbapenemase coding genes VIM and NDM were never detected (Table 5).

The bacterial isolates from sediments (Table 6) always carried the ESBL-coding gene CTX-M 1, while other studied genes were absent. Plasmid-mediated quinolone-resistance gene *qnr S* was found in bacterial isolates from sediments in autumn and winter, while sulfonamide resistance coding genes *sul1, sul2* were present in them in all seasons; carbapenemase coding genes VIM and NDM were never detected. 

When phylogenetic grouping was analyzed, the ESBL-producing *E. coli* isolates from river water and sediment were found to belong to phylogenetic groups A, B1, B2, and D. In the case of river water, the number of isolates that belonged to A and B1 was more than those in groups B2 and D and a significant seasonal variation in B2 group (*p* < 0.05) was observed. A group was predominant in the case of bacterial isolates from sediment (Table 4 and Table 5).

## 4. Discussion

To our knowledge, this is the first comprehensive, seasonal variation study over a period of one year focusing on antibiotic residues, resistance, resistance genes and water quality in a river in India. 

We found significant seasonal variation among different water quality parameters. Sulfamethoxazole concentrations were significantly associated with seasonal variation in pH of river water. Significant seasonal variation was found in river water *E. coli* resistance to ampicillin, cefepime, meropenem, amikacin, gentamicin, tigecycline, and in multidrug resistance. In *E. coli* from sediment, meropenem resistance was associated significantly with seasonal variation. Significant seasonal variation was found in the detection of *bla*CTX-M-1 gene and majority of the ESBL-producing *E. coli* isolates from river water and sediment in all different seasons belonged to phylogenetic group A and B1.

The Kshipra River is used for many worship rituals and various activities leading to the depletion of water quality, and this changes the ecology of the river. The present study showed significant seasonal variation among different water quality parameters. *E. coli* and total coliforms have been used as fecal indicator organisms for microbial source tracking [40,41]. Total coliforms and *E. coli* levels were higher than the levels permitted for different types of water [42]. An earlier study found that mass bathing had a negative impact on the water quality [30], with increased values of different microbial and physico-chemical parameters during and after mass bathes. For instance, contents of total coliform, *E. coli* were found to be high after a mass-bathing event [43]. The results of water quality parameters revealed that the river water is not suitable for drinking and bathing purposes (Table S2). 

Sulfamethoxazole was detected in river water in all seasons with higher levels in autumn and winter than in summer and rainy season, but in sediment it was found in winter season only. Sulfonamides are not readily biodegradable [44] but are characterized to have high water solubility and low sorption coefficient [44,45,46]. Photo-degradation is considered an important degradation pathway of sulfonamides [47]. Various factors can facilitate the degradation of antibiotics in summer including; rise in temperature, photolysis and microbial activity. Additionally, addition of the wastewater and promotion the movement of target antibiotics in rainy season may also contribute [48,49]. The observed higher values of antibiotic residues in the river samples in winter and autumn can clearly be assigned for all above factors. The current results showed significant association between season and water quality parameters, including pH and sulfamethoxazole (*p* < 0.05). It has been shown in earlier studies that sorption of sulfonamides is strongly dependent on pH [50,51]. While in some studies sulfonamides was classified as resistant to biodegradation [52], and high proportion of sulfonamides is bound to the soil as non-extractable residues [53] were found with a low bioavailability to microorganisms [54]. Sulfonamides are likely to occur in a bioavailable form in the environment based on their physiochemical properties and relative stability and may therefor pose a risk for development of resistance to antibiotics. In a study in the Cache La Poudre River, US, the highest levels of sulfamethoxazole were found in winter [13]. In a study from the Jiulongjiang River Basin, South China, concentration of sulfamethoxazole was higher in winter compared to summer [55].

Norfloxacin and ofloxacin were found in river water only in autumn, and ofloxacin was detected in sediment only in winter. Most fluoroquinolones show poor water solubility at pH 6–8 and low susceptibility to microbial degradation at increased temperatures [56,57]. Furthermore, fluoroquinolones have high sorption potential [58] and high persistence in soil and sediments. Reduction of 88−92% of fluoroquinolones in wastewater treatment was reported, mainly due to strong sorption on sewage sludge [59]. Other studies have found seasonal variation in release of antibiotics in the environment, one study conducted in Ujjain, India, reported that the highest concentrations of fluoroquinolones were released in hospital waste water in winter followed by rainy season and summer [32]. Enrofloxacin and ofloxacin in surface water presented higher residual concentrations in summer than those in rains in water samples from the Yellow River Delta, China [60]. In another study, concentrations of ofloxacin in winter were higher than those in summer [55]. Total residual antibiotic as β-lactam was found in river water only in summer and in sediment only in rainy season. β-lactams are considered less susceptible to adsorption, they can be readily degraded [57] and are probably the most susceptible antibiotic to hydrolysis [49], resulting in less persistence in the environment. Some studies showed the occurrence of β-lactam antibiotic residues in the environment despite their relative poor stability. A study that analyzed β-lactam residues in water samples taken at 16 river banks in north Rhine-Westphalia, Germany, found very low levels of β-lactams [61]. Another study reported similar results for water samples of the Poudre River in northern Colorado, USA [62]. 

Antibiotic pollution is likely to lead to the development of antibiotic resistant bacteria/resistance genes in the aquatic environment [63]. The current results showed presence of antibiotic-resistant *E. coli* in river water and sediment. The percentage of resistance to ciprofloxacin, nalidixic acid, tetracycline, sulfamethiazole, and multidrug resistance was found to be highest in autumn in both river water and sediment. The resistance to imipenem, meropenem, amikacin, gentamicin, and tigecycline was higher in winter in both river water and sediment than in other seasons. The majority of the ESBL-producing *E. coli* isolates from river water and sediment belonged to phylogenetic groups A and B1 to which commensal *E. coli* are classified [39]. Antibiotic resistance among *E coli* is of increasing global concern. This has been associated with the spread of extended spectrum β-lactamase producing *E. coli* [64,65]. Growing evidence suggests that antibiotic resistant bacteria/resistance genes are increasing in the environment associated with the discharges from domestic pharmaceutical manufacturing and hospital wastewater [66,67], agricultural use and releases [68], and other causes [69,70]. Antibiotic resistant bacteria/resistance genes in sediments are acquired from water environments. River sediments are important antibiotic resistance reservoirs where various antibiotic resistant bacteria/resistance genes are concentrated [71]. The occurrence and resistance to antibiotics in water and sediment in different seasons is mainly influenced by the physicochemical and environmental factors (pH, temperature, sorption, degradation etc.) [46,47]. For instance, β-lactamase are easily hydrolyzed at ambient temperature [72] while, quinolones and tetracyclines are susceptible to photodegradation [73,74]. Other factors include, widespread use and patterns of antibiotics that accelerate the development and the selection of antibiotic resistance genes [75,76,77]. In addition, the frequency of antibiotic use for different purposes during different periods, results in variation of antibiotic concentrations in different seasons and environmental compartments [78]. Moreover, studies have shown that the presence of antibiotics at low sub-inhibitory level can accelerate horizontal transfer of environmental antibiotic resistance genes [14]. Exposures to very low levels of antibiotic concentrations below the minimal inhibitory concentration (MIC) are sufficient to maintain antibiotic resistance [21,79]. Our other studies conducted in the same geographical area, as this paper, showed antibiotic resistance of *E. coli* isolates from most children, to at least one antibiotic and co-resistance to cephalosporins and quinolones [80], similar patterns of antibiotic resistance and multidrug resistance in commensal bacteria from humans and their environment [15] and antibiotic resistance to cephalosporins, quinolones, and imipenem of *E. coli* isolates from hospital wastewater [37]. Other studies have found resistance to aminoglycoside, sulfonamide, and trimethoprim in river water in India [81,82], *sul* genes in a natural river basin of China [83], *Sul1* and *sul2* in river water from Colorado, USA [68].

The present results showed variation in water quality parameters (physical, chemical and microbiological) and pollution of water by antibiotics and antibiotic resistant bacteria/resistance genes in case of the Kshipra River in India in different seasons and this water if used without proper treatment can lead to various health hazards. 

Antibiotics were originally produced by microorganisms, but the definition has been extended to synthetic and semi-synthetic compounds with similar properties [84]. In general, in environmental studies [1,6,8,14,32,44,57,63] and even otherwise in many cases in literature, the term is loosely used to refer to all antibacterial compounds.

## 5. Conclusions

Antibiotic pollution and resistance, and resistance genes were found in water of the Kshipra River in India during various seasons of a year. In river water, seasonal variation was significantly associated with different water quality parameters, concentrations of sulfamethoxazole, bacteria resistant to ampicillin, cefepime, meropenem, amikacin, gentamicin, tigecycline, and multidrug resistance and the resistance gene CTX-M-1. In sediment, meropenem resistance was associated significantly with seasonal variation. Majority of the ESBL-producing *E. coli* isolates from river water and sediment in all different seasons belonged to phylogenetic group A and B1. Studies are needed to examine the fate of antibiotic residues and to assess the risk of their effect on resistance in all environmental compartments, to set up regulatory standards by the authorities and to control the dissemination of these “pollutants” in the environment.

## Figures and Tables

**Table 1:** Concentrations of antibiotic residues measured in different seasons in waters of the Kshipra River in India.

River Water Samples									
Antibiotic	Summer (*N* = 14)		Rain (*N* = 14)		Autumn (*N* = 14)		Winter (*N* = 14)		*p*-Value
	*n*	Mean (Range) µg/L	*n*	Mean (Range) µg/L	*n*	Mean (Range) µg/L	*n*	Mean (Range) µg/L	
Ceftriaxone	0	BDL	0	BDL	0	BDL	0	BDL	-
Ciprofloxacin	0	BDL	0	BDL	0	BDL	0	BDL	-
Norfloxacin	0	BDL	0	BDL	6	0.66 (0–0.98)	0	BDL	-
Ofloxacin	0	BDL	0	BDL	7	0.99 (0.64–1.46)	0	BDL	-
Metronidazole	0	BDL	0	BDL	0	BDL	0	BDL	-
Sulfamethoxazole	14	1.39 (0.24–2.21)	4	0.04 (0–0.17)	7	2.75 (1.05–4.66)	7	2.18 (0.5–3.47)	<0.0001 *
*Total Residual Antibiotics as Beta Lactam*	*n* (%)		*n* (%)		*n* (%)		*n* (%)		
Present (>5 ppb)	7 (50)		0 (0)		0 (0)		0 (0)		-

Abbreviations: BDL; below detection limit. Note: *p*-value are extracted from ANOVA; *N* = number of samples collected; *n* = number of samples with detected antibiotic residues. * adjusted for pH, water temperature, Carbonate Alkalinity, Bicarbonate Alkalinity, Chloride, Nitrate Nitrogen, Total Phosphorus, Total Coliform and Total *E. coli*.

**Table 2:** Concentrations of antibiotic residues measured in different seasons in sediment of the Kshipra River in India.

River Sediment Samples									
Antibiotic	Summer (*N* = 0)		Rain (*N* = 7)		Autumn (*N* = 7)		Winter (*N* = 14)		*p*-Value
	*n*	Mean (Range) µg/L	*n*	Mean (Range) µg/L	*n*	Mean (Range) µg/L	*n*	Mean (Range) µg/L	
Ceftriaxone	-	-	BDL	BDL	BDL	BDL	BDL	BDL	-
Ciprofloxacin	-	-	BDL	BDL	BDL	BDL	BDL	BDL	-
Norfloxacin	-	-	BDL	BDL	BDL	BDL	BDL	BDL	-
Ofloxacin	-	-	BDL	BDL	BDL	BDL	1	1.39 (0–9.74)	-
Metronidazole	-	-	BDL	BDL	BDL	BDL	BDL	BDL	-
Sulfamethoxazole	-	-	BDL	BDL	BDL	BDL	1	1.18 (0–8.23)	-
*Total Residual Antibiotics as Beta Lactam*	*n* (%)		*n* (%)		*n* (%)		*n* (%)		
Present (>5 ppb)	-		4 (57.1)		0		0		-

Abbreviations: BDL; below detection limit. Note: *p*-value are extracted from ANOVA; *N* = number of samples collected; *n* = number of samples with detected antibiotic residues.

**Table 3:** Antibiotic resistance and multidrug resistance patterns of *E. coli* isolated from river water samples in different seasons from the Kshipra River in India.

River Water Samples					
Antibiotic	Summer *N* = 70 *	Rain *N* = 80 **	Autumn *N* = 70 ***	Winter *N* = 83 ****	*p*-Value
	*n* (%)	*n* (%)	*n* (%)	*n* (%)	
Ampicillin	12 (17)	27 (33)	32 (45)	33 (39)	0.002
Cefotaxime	14 (20)	15 (18)	14 (20)	24 (28)	0.4
Ceftazidime	10 (14)	12 (15)	15 (21)	13 (15)	0.7
Cefepime	0 (0)	6 (7)	10 (14)	9 (10)	0.004
Ceftriaxone	9 (12)	-	-	-	-
Imipenem	1 (1)	1 (1)	1 (1)	4 (4)	0.5
Meropenem	7 (10)	0 (0)	4 (5)	22 (26)	<0.0001
Ciprofloxacin	4 (5)	7 (8)	10 (14)	12 (14)	0.2
Nalidixic acid	8 (11)	11 (13)	16 (22)	21 (25)	0.08
Amikacin	13 (15)	0 (0)	0 (0)	13 (15)	<0.0001
Gentamicin	0 (0)	2 (2)	1 (1)	8 (9)	0.007
Nitrofurantoin	0 (0)	3 (3)	3 (4)	5 (6)	0.2
Tetracycline	3 (4)	10 (12)	12 (17)	11 (13)	0.09
Tigecycline	0 (0)	0 (0)	0 (0)	7 (8)	0.0002
Sulfamethizole	5 (7)	11 (13)	12 (17)	7 (8)	0.2
Co-trimoxazole	4 (5)	11 (13)	11 (15)	9 (10)	0.3
Colistin	0 (00)	-	-	-	-
ESBL	7 (8)	10 (11)	10 (12)	5 (6)	0.4
MDR	2 (2)	15 (17)	19 (24)	20 (23)	<0.0001

Abbreviations: ESBL; Extended Spectrum Beta-Lactamase, MDR; multidrug resistance. Note: *p*-value are extracted from Fisher test; *N* = number of samples detected; *n*: =number of test bacterial isolates; * *n* = 84 for MDR and ESBL; ** *n* = 0 for colistin and ceftriaxone, *n* = 84 for MDR and ESBL; *** *n* = 0 for colistin and ceftriaxone, *n* = 78 for MDR and ESBL; **** *n* = 0 for colistin and ceftriaxone, *n* = 84 for MDR and ESBL; Not done.

**Table 4:** Antibiotic resistance and multidrug resistance patterns of *E. coli* isolated from river sediment samples in different seasons from the Kshipra River in India.

Sediment Samples				
Antibiotics	Rain *N* = 31 *	Autumn *N* = 27 **	Winter *N* = 39 ***	*p*-Value
	*n* (%)	*n* (%)	*n* (%)	
Ampicillin	9 (29)	9 (33)	12 (30)	0.96
Cefotaxime	9 (29)	6 (22)	18 (46)	0.12
Ceftazidime	9 (29)	6 (22)	8 (20)	0.76
Cefepime	6 (20)	2 (7)	6 (15)	0.41
Imipenem	0 (0)	0 (0)	2 (5)	0.33
Meropenem	0 (0)	0 (0)	11 (28)	<0.0001
Ciprofloxacin	6 (19)	7 (25)	6 (15)	0.62
Nalidixic acid	7 (22)	11 (40)	11 (28)	0.34
Amikacin	0 (0)	1 (3)	2 (5)	0.62
Gentamicin	0 (0)	0 (0)	2 (5)	0.33
Nitrofurantoin	2 (6)	0 (0)	0 (0)	0.18
Tetracycline	3 (9)	8 (29)	7 (17)	0.15
Tigecycline	0 (0)	0 (0)	2 (5)	0.33
Sulfamethizole	5 (16)	7 (25)	7 (17)	0.65
Co-trimoxazole	6 (19)	8 (29)	7 (17)	0.55
ESBL	9 (26)	4 (13)	7 (17)	0.49
MDR	9 (26)	10 (34)	10 (24)	0.67

Abbreviations: ESBL; Extended Spectrum Beta-Lactamase, MDR; multidrug resistance. Note: *p*-value are extracted from Fisher test; *N* = number of samples detected; *n*: number of test bacterial isolates; * *n* = 30 for cefepime and *n* = 34 for ESBL and MDR; ** *n* = 29 for ESBL and MDR; *** *n* = 41 for ESBL and MDR.

**Table 5:** Antibiotic resistance-coding genes present in the *Escherichia coli* isolates from river water in different seasons in the Kshipra River in India and their phylogenic groups.

River Water Samples					
Antibiotic Resistance Genes	Summer	Rain	Autumn	Winter	*p*-Value
	*n* (%)	*n* (%)	*n* (%)	*n* (%)	
CTX-M-1 ^a^	8 (23)	10 (31)	9 (50)	8 (19)	0.09
CTX-M-2 ^a^	0 (0)	0 (0)	0 (0)	0 (0)	-
CTX-M-9 ^a^	0 (0)	0 (0)	0 (0)	0 (0)	-
*qnrA* ^b^	0 (0)	0 (0)	0 (0)	0 (0)	-
*qnrB* ^b^	(5)	0 (0)	0 (0)	0 (0)	0.2
*qnrS* ^b^	2 (10)	6 (23)	2 (8)	7 (16)	0.5
*Sul I* ^c^	2 (5)	4 (12)	3 (13)	4 (12)	0.6
*Sul II* ^c^	4 (10)	6 (18)	5 (21)	4 (12)	0.6
NDM ^d^	0 (0)	0 (0)	0 (0)	0 (0)	-
VIM ^d^	0 (0)	0 (0)	0 (0)	0 (0)	-
Phylogenic groups					
A ^e^	30 (63)	38 (66)	28 (60)	34 (52)	0.5
B1 ^e^	14 (30)	11 (19)	15 (32)	16 (25)	0.4
B2 ^e^	0 (0)	2 (3)	0 (0)	6 (9)	0.03
D ^e^	4 (8)	5 (9)	4 (9)	9 (14)	0.8

*p*-value are extracted from Fisher test. ^a^ samples: Autumn *n* = 18, Rain *n* = 32, Summer *n* = 35 and Winter *n* = 42; ^b^ samples: Autumn *n* = 25, Rain *n* = 26, Summer *n* = 20 and Winter *n* = 43; ^c^ samples: Autumn *n* = 24, Rain *n* = 33, Summer *n* = 40 and Winter *n* = 33; ^d^ samples: Autumn *n* = 14, Rain *n* = 12, Summer *n* = 18 and Winter *n* = 33; ^e^ samples: Autumn *n* = 47, Rain *n* = 58, Summer *n* = 48 and Winter *n* = 65.

**Table 6:** Antibiotic resistance-coding genes present in the Escherichia coli isolates from river sediment in different seasons in the Kshipra River in India.

Sediment Samples				
Antibiotic Resistance Genes	Rain	Autumn	Winter	*p*-Value
	*n* (%)	*n* (%)	*n* (%)	
CTX-M-1 ^a^	5 (25)	2 (40)	7 (41)	0.6
CTX-M-2 ^a^	0 (0)	0 (0)	0 (0)	-
CTX-M-9 ^a^	0 (0)	0 (0)	0 (0)	-
*qnr* A ^b^	0 (0)	0 (0)	0 (0)	-
*qnr* B	0 (0)	0 (0)	0 (0)	-
*qnr* S ^b^	0 (0)	2 (15)	4 (44)	0.3
*sul I* ^c^	1 (25)	3 (33)	2 (33)	0.9
*sul II* ^c^	1 (25)	3 (33)	4 (67)	0.5
NDM ^d^	0 (0)	0 (0)	0 (0)	-
VIM ^d^	0 (0)	0 (0)	0 (0)	-
Phylogenic groups				
A ^e^	16 (76)	11 (56)	15 (63)	0.5
B1 ^e^	4 (19)	2 (11)	5 (21)	0.7
B2 ^e^	0 (0)	1 (5)	0 (0)	0.3
D ^e^	1 (5)	4 (21)	4 (17)	0.3

*p*-values are extracted from Fisher test; ^a^ samples: Autumn *n* = 5, Rain *n* = 20 and Winter *n* = 17; ^b^ samples: Autumn *n* = 13, Rain *n* = 3 and Winter *n* = 9; ^c^ samples: Autumn *n* = 9, Rain *n* = 4 and Winter *n* = 6; ^d^ samples: Autumn *n* = 6, Rain *n* = 6 and Winter *n* = 17; ^e^ samples: Autumn *n* = 19, Rain *n* = 21 and Winter *n* = 24.

## Supplementary Materials

The following are available online at http://www.mdpi.com/1660-4601/15/6/1281/s1, Table S1: Average value of water quality parameter. Table S2: Water Quality Criteria.

## References

[1] Gotha R., Shashidhar T. (2015). Antibiotic Pollution in the Environment: A Review. Clean Soil Air Water.

[2] Jjemba P.K. (2006). Excretion and ecotoxicity of pharmaceutical and personal care products in the environment. Ecotoxicol. Environ. Saf..

[3] Lienert J., Bürki T., Escher B.I. (2007). Reducing micropollutants with source control: Substance flow analysis of 212 pharmaceuticals in faeces and urine. Water Sci. Technol. J. Int. Assoc. Water Pollut. Res..

[4] Boxall A.B.A., Kolpin D.W., Halling-Sørensen B., Tolls J. (2003). Are veterinary medicines causing environmental risks?. Environ. Sci. Technol..

[5] Davis J.G., Truman C.C., Kim S.C., Ascough J.C., Carlson K. (2006). Antibiotic transport via runoff and soil loss. J. Environ. Qual..

[6] Kümmerer K. (2009). Antibiotics in the aquatic environment—A review—Part I. Chemosphere.

[7] Miao X.-S., Bishay F., Chen M., Metcalfe C.D. (2004). Occurrence of antimicrobials in the final effluents of wastewater treatment plants in Canada. Environ. Sci Technol..

[8] Chang X., Meyer M.T., Liu X., Zhao Q., Chen H., Chen J., Qiu Z., Yang L., Cao J., Shu W. (2010). Determination of antibiotics in sewage from hospitals, nursery and slaughter house, wastewater treatment plant and source water in Chongqing region of Three Gorge Reservoir in China. Environ. Pollut. Barking Essex.

[9] Tamtam F., Mercier F., Le Bot B., Eurin J., Tuc Dinh Q., Clément M., Chevreuil M. (2008). Occurrence and fate of antibiotics in the Seine River in various hydrological conditions. Sci. Total Environ..

[10] Zhou L.-J., Ying G.-G., Zhao J.-L., Yang J.-F., Wang L., Yang B., Liu S. (2011). Trends in the occurrence of human and veterinary antibiotics in the sediments of the Yellow River, Hai River and Liao River in northern China. Environ. Pollut. Barking Essex.

[11] Hari A.C., Paruchuri R.A., Sabatini D.A., Kibbey T.C.G. (2005). Effects of pH and cationic and nonionic surfactants on the adsorption of pharmaceuticals to a natural aquifer material. Environ. Sci. Technol..

[12] Beausse J. (2004). Selected Drugs in solid matrices: A review of environmental determination, occurrence and properties of principal substances. TrAC Trends Anal. Chem..

[13] Kim S.-C., Carlson K. (2007). Temporal and spatial trends in the occurrence of human and veterinary antibiotics in aqueous and river sediment matrices. Environ. Sci. Technol..

[14] Kümmerer K. (2004). Resistance in the environment. J. Antimicrob. Chemother..

[15] Purohit M.R., Chandran S., Shah H., Diwan V., Tamhankar A.J., Stålsby Lundborg C. (2017). Antibiotic resistance in an Indian rural community: A ”One-Health” observational study on commensal coliform from humans, animals, and water. Int. J. Environ. Res. Public Health.

[16] Bailey J.K., Pinyon J.L., Anantham S., Hall R.M. (2010). Commensal *Escherichia coli* of healthy humans: A reservoir for antibiotic-resistance determinants. J. Med. Microbiol..

[17] Edge T.A., Hill S. (2005). Occurrence of antibiotic resistance in *Escherichia coli* from surface waters and fecal pollution sources near Hamilton, Ontario. Can. J. Microbiol..

[18] Watkinson A.J., Micalizzi G.B., Graham G.M., Bates J.B., Costanzo S.D. (2007). Antibiotic-resistant *Escherichia coli* in wastewaters, surface waters, and oysters from an urban riverine system. Appl. Environ. Microbiol..

[19] Zhang X.-X., Zhang T., Fang H.H.P. (2009). Antibiotic resistance genes in water environment. Appl. Microbiol. Biotechnol..

[20] Auerbach E.A., Seyfried E.E., McMahon K.D. (2007). Tetracycline resistance genes in activated sludge wastewater treatment plants. Water Res..

[21] Gullberg E., Albrecht L.M., Karlsson C., Sandegren L., Andersson D.I. (2014). Selection of a multidrug resistance plasmid by sublethal levels of antibiotics and heavy metals. mBio.

[22] WHO (2008). Communicable Disease Alert and Response for Mass Gatherings. http://www.who.int/csr/Mass_gatherings2.pdf?ua=1.

[23] Fick J., Söderström H., Lindberg R.H., Phan C., Tysklind M., Larsson D.G.J. (2009). Contamination of surface, ground, and drinking water from pharmaceutical production. Environ. Toxicol. Chem..

[24] Larsson D.G.J., de Pedro C., Paxeus N. (2007). Effluent from drug manufactures contains extremely high levels of pharmaceuticals. J. Hazard. Mater..

[25] Ramaswamy B.R., Shanmugam G., Velu G., Rengarajan B., Larsson D.G.J. (2011). GC-MS analysis and ecotoxicological risk assessment of triclosan, carbamazepine and parabens in Indian rivers. J. Hazard. Mater..

[26] Gotha R., Thatikonda S. (2017). Mathematical model for the transport of fluoroquinolone and its resistant bacteria in aquatic environment. Environ. Sci. Pollut. Res. Int..

[27] Farook Ahmed T., Sushil M., Krishna M. (2012). Impact of Dye Industrial effluent on physicochemical characteristics of Kshipra river, Ujjain city, India. Int. Res. J. Environ. Sci..

[28] Gupta R.C., Gupta A.K., Shrivastava R.K. (2012). Assessment of water quality status of holy river Kshipra using water quality index. J. Indian Water Resour. Soc..

[29] David S., Roy N. (2016). Public health perspectives from the biggest human mass gathering on earth: Kumbh mela, India. Int. J. Infect. Dis..

[30] Pawar R.S., Bhatia R.K., Pawar R.S., Bhatia R.K. (2016). Assessment of Water Quality of River Kshipra during Simhastha Mahakumbh Mela 2016 in Ujjain, Madhya Pradesh. Int. J. Innov. Res. Sci. Technol..

[31] Diwan V., Purohit M., Chandran S., Parashar V., Shah H., Mahadik V.K., Lundborg C.S., Tamhankar A.J. (2017). A three-year follow-up study of antibiotic and metal residues, antibiotic resistance and resistance genes, focusing on Kshipra-a river associated with holy religious mass-bathing in India: Protocol paper. Int. J. Environ. Res. Public Health.

[32] Diwan V., Stålsby Lundborg C., Tamhankar A.J. (2013). Seasonal and temporal variation in release of antibiotics in hospital wastewater: Estimation using continuous and grab sampling. PLoS ONE.

[33] Diwan V., Tamhankar A.J., Khandal R.K., Sen S., Aggarwal M., Marothi Y. (2010). Antibiotics and antibiotic-resistant bacteria in waters associated with a hospital in Ujjain, India. BMC Public Health.

[34] Huang C.-H., Renew J., Smeby K., Pinkston K., Sedlak D. (2011). Assessment of potential antibiotic contaminants in water and preliminary occurrence analysis. J. Contemp. Water Res. Educ..

[35] Nataro J., Bopp C., Fields P., Kaper J., Strockbine N. (2007). Escherichia, Shigella, and Salmonella. Manual of Clinical Microbiology.

[36] CLSI (2014). Performance Standards for Antimicrobial Susceptibility Testing; Twenty-Fourth Informational Supplement. Clin. Lab. Stand. Inst..

[37] Chandran S.P., Diwan V., Tamhankar A.J., Joseph B.V., Rosales-Klintz S., Mundayoor S., Lundborg C.S., Macaden R. (2014). Detection of carbapenem resistance genes and cephalosporin, and quinolone resistance genes along with oqxAB gene in *Escherichia coli* in hospital wastewater: A matter of concern. J. Appl. Microbiol..

[38] Titilawo Y., Obi L., Okoh A. (2015). Antimicrobial resistance determinants of *Escherichia coli* isolates recovered from some rivers in Osun State, South-Western Nigeria: Implications for public health. Sci. Total Environ..

[39] Clermont O., Bonacorsi S., Bingen E. (2000). Rapid and simple determination of the *Escherichia coli* phylogenetic group. Appl. Environ. Microbiol..

[40] Ashbolt N.J., Grabow W.O., Snozzi M. (2001). Indicators of Microbial Water Quality.

[41] Ishii S., Sadowsky M.J. (2008). *Escherichia coli* in the environment: Implications for water quality and human health. Microbes Environ..

[42] EPA (2013). Revised Total Coliform Rule (RTCR): A Quick Reference Guide. https://nepis.epa.gov/Exe/ZyPDF.cgi?Dockey=P100K9MP.txt.

[43] Bhasin S., Shukla A.N., Shrivastava S. (2015). Impact of mass bathing on water quality of river Kshipra at Triveni, Ujjain, M.P. India. Int. J. Adv. Life Sci..

[44] Ingerslev F., Halling-Sorensen B. (2000). Biodegradability properties of sulfonamides in activated sludge. Environ. Toxicol. Chem..

[45] Boxall A.B.A., Blackwell P., Cavallo R., Kay P., Tolls J. (2002). The sorption and transport of a sulphonamide antibiotic in soil systems. Toxicol. Lett..

[46] Tolls J. (2001). Sorption of veterinary pharmaceuticals in soils: A review. Environ. Sci. Technol..

[47] Beek B. (2001). Biodegradation and Persistence.

[48] Alexy R., Kümpel T., Kümmerer K. (2004). Assessment of degradation of 18 antibiotics in the closed bottle test. Chemosphere.

[49] Huang M., Braselton W.E., Rumbeiha W.K., Johnson M. (2011). Rapid and reliable identification of ionophore antibiotics in feeds by liquid chromatography-tandem mass spectrometry. J. Vet. Diagn Investig. Off. Publ. Am. Assoc. Vet. Lab. Diagn Inc..

[50] Gao J., Pedersen J.A. (2005). Adsorption of sulfonamide antimicrobial agents to clay minerals. Environ. Sci. Technol..

[51] Kahle M., Stamm C. (2007). Time and pH-dependent sorption of the veterinary antimicrobial sulfathiazole to clay minerals and ferrihydrite. Chemosphere.

[52] Al-Ahmad A., Daschner F.D., Kummerer K. (1999). biodegradability of cefotiam, ciprofloxacin, meropenem, penicillin G, and sulfamethoxazole and inhibition of waste water bacteria. Arch. Environ. Contam. Toxicol..

[53] Höltge S., Kreuzig R. (2007). Laboratory Testing of sulfamethoxazole and its metabolite acetyl-sulfamethoxazole in Soil. Clean Soil Air Water.

[54] Heise J., Höltge S., Schrader S., Kreuzig R. (2006). Chemical and biological characterization of non-extractable sulfonamide residues in soil. Chemosphere.

[55] Zhang H., Du M., Jiang H., Zhang D., Lin L., Ye H., Zhang X. (2015). Occurrence, seasonal variation and removal efficiency of antibiotics and their metabolites in wastewater treatment plants, Jiulongjiang River Basin, South China. Environ. Sci. Process. Impacts..

[56] Ternes T.A. (2001). Analytical methods for the determination of pharmaceuticals in aqueous environmental samples. TrAC Trends Anal. Chem..

[57] Thiele-Bruhn S. (2003). Pharmaceutical antibiotic compounds in soils—A review. J. Plant. Nutr. Soil Sci..

[58] Picó Y., Andreu V. (2007). Fluoroquinolones in soil—Risks and challenges. Anal. Bioanal. Chem..

[59] Golet E.M., Strehler A., Alder A.C., Giger W. (2002). Determination of fluoroquinolone antibacterial agents in sewage sludge and sludge-treated soil using accelerated solvent extraction followed by solid-phase extraction. Anal. Chem..

[60] Zhao S., Liu X., Cheng D., Liu G., Liang B., Cui B., Bai J. (2016). Temporal-spatial variation and partitioning prediction of antibiotics in surface water and sediments from the intertidal zones of the Yellow River Delta, China. Sci. Total Environ..

[61] Christian T., Schneider R.J., Färber H.A., Skutlarek D., Meyer M.T., Goldbach H.E. (2003). determination of antibiotic residues in manure, soil, and surface waters. Acta Hydrochim. Hydrobiol..

[62] Cha J.M., Yang S., Carlson K.H. (2006). Trace determination of beta-lactam antibiotics in surface water and urban wastewater using liquid chromatography combined with electrospray tandem mass spectrometry. J. Chromatogr. A.

[63] Martínez J.L. (2008). Antibiotics and antibiotic resistance genes in natural environments. Science.

[64] Guenther S., Ewers C., Wieler L.H. (2011). Extended-spectrum beta-lactamases producing *E. coli* in wildlife, yet another form of environmental pollution?. Front. Microbiol..

[65] Peirano G., van Greune C.H.J., Pitout J.D.D. (2011). Characteristics of infections caused by extended-spectrum β-lactamase-producing *Escherichia coli* from community hospitals in South Africa. Diagn. Microbiol. Infect. Dis..

[66] Iwane T., Urase T., Yamamoto K. (2001). Possible impact of treated wastewater discharge on incidence of antibiotic resistant bacteria in river water. Water Sci. Technol. J. Int. Assoc. Water Pollut. Res..

[67] Li D., Yang M., Hu J., Zhang J., Liu R., Gu X., Zhang Y., Wang Z. (2009). Antibiotic-resistance profile in environmental bacteria isolated from penicillin production wastewater treatment plant and the receiving river. Environ. Microbiol..

[68] Pei R., Kim S.-C., Carlson K.H., Pruden A. (2006). Effect of river landscape on the sediment concentrations of antibiotics and corresponding antibiotic resistance genes (ARG). Water Res..

[69] Séveno N.A., Kallifidas D., Smalla K., van Elsas J.D., Collard J.M., Karagouni A.D., Wellington E.M. (2002). Occurrence of reservoirs of antibiotic resistance genes in the environment. Rev. Med. Microbiol..

[70] Knapp C.W., Dolfing J., Ehlert P.A.I., Graham D.W. (2010). Evidence of increasing antibiotic resistance gene abundances in archived soils since 1940. Environ. Sci. Technol..

[71] Kristiansson E., Fick J., Janzon A., Grabic R., Rutgersson C., Weijdegård B. (2011). Pyrosequencing of antibiotic-contaminated river sediments reveals high levels of resistance and gene transfer elements. PLoS ONE.

[72] Längin A., Alexy R., König A., Kümmerer K. (2009). Deactivation and transformation products in biodegradability testing of beta-lactams amoxicillin and piperacillin. Chemosphere.

[73] Davies A.K., McKellar J.F., Phillips G.O., Reid A.G. (1979). Photochemical oxidation of tetracycline in aqueous solution. J. Chem. Soc. Perkin Trans..

[74] Torniainen K., Tammilehto S., Ulvi V. (1996). The effect of pH, buffer type and drug concentration on the photodegradation of ciprofloxacin. Int. J. Pharm..

[75] Allen H.K., Donato J., Wang H.H., Cloud-Hansen K.A., Davies J., Handelsman J. (2010). Call of the wild: Antibiotic resistance genes in natural environments. Nat. Rev. Microbiol..

[76] Schjørring S., Krogfelt K.A. (2011). Assessment of bacterial antibiotic resistance transfer in the gut. Int. J. Microbiol..

[77] Sørensen S.J., Bailey M., Hansen L.H., Kroer N., Wuertz S. (2005). Studying plasmid horizontal transfer in situ: A critical review. Nat. Rev. Microbiol..

[78] Sun L., Klein E.Y., Laxminarayan R. (2012). Seasonality and temporal correlation between community antibiotic use and resistance in the United States. Clin. Infect. Dis. Off. Publ. Infect. Dis. Soc. Am..

[79] Gullberg E., Cao S., Berg O.G., Ilbäck C., Sandegren L., Hughes D. (2011). Selection of resistant bacteria at very low antibiotic concentrations. PLoS Pathog..

[80] Shakya P., Barrett P., Diwan V., Marothi Y., Shah H., Chhari N. (2013). Antibiotic resistance among *Escherichia coli* isolates from stool samples of children aged 3 to 14 years from Ujjain, India. BMC Infect. Dis..

[81] Mohapatra H., Mohapatra S.S., Mantri C.K., Colwell R.R., Singh D.V. (2008). Vibrio cholerae non-O1, non-O139 strains isolated before 1992 from Varanasi, India are multiple drug resistant, contain intSXT, dfr18 and aadA5 genes. Environ. Microbiol..

[82] Mukherjee S., Chakraborty R. (2006). Incidence of class 1 integrons in multiple antibiotic-resistant Gram-negative copiotrophic bacteria from the River Torsa in India. Res. Microbiol..

[83] Hu J., Shi J., Chang H., Li D., Yang M., Kamagata Y. (2008). Phenotyping and genotyping of antibiotic-resistant *Escherichia coli* isolated from a natural river basin. Environ. Sci. Technol..

[84] Dorland W.A.N. (2007). Dorland’s Illustrated Medical Dictionary.

