# Supplementary material for: Seasonal Variations in Water-Quality, Antibiotic Residues, Resistant Bacteria and Antibiotic Resistance Genes of Escherichia coli Isolates from Water and Sediments of the Kshipra River in Central India
Source: Int J Environ Res Public Health. 2018 Jun 17;15(6):1281. doi: 10.3390/ijerph15061281 (PMC6024939; doi:10.3390/ijerph15061281)
Supplement: Supplementary file 1 [file ijerph-15-01281-s001.pdf]

Supplementary materials

Table S1. Average value of water quality parameter.

Water Quality Parameter	Autumn Average (sd)	Summer Average (sd)	Rain Average (sd)	Winter Average (sd)	P-value
pH	8.5 (0.24)	8.3 (0.3)	8.3 (0.1)	8.6 (0.3)	<0.0001
Air Temp.(°C)	31.4 (2.9)	39.3 (0.9)	29.1 (1.4)	21.6 (3.4)	<0.0001
Water Temp. (°C)	29.4 (1.6)	32.9 (1)	28.7 (0.5)	19.4 (0.7)	<0.0001
Conductivity (μS/cm)	1199.9 (70.9)	1328.8 (65.7)	1368.8 (83.3)	1375.8 (82)	<0.0001
TDS (mg/l)	742.7 (83.5)	816.7 (48.9)	835 (50.8)	889.3 (33.5)	<0.0001
Turbidity(NTU)	16.3 (5)	76 (21.3)	-	40.1 (43.4)	<0.0001
Total Suspended Solids (mg/l)	73.7 (27.7)	81.7 (25.1)	87.7 (24.4)	78 (52)	0.06
Carbonate Alkalinity(mg/l)	108.3 (54.2)	95.3 (51.9)	112 (47.7)	98.6 (56.8)	0.14
Bicarbonate Alkalinity(mg/l)	37.2 (18.6)	32.7 (15.6)	38.9 (15.8)	35.7 (18.2)	0.12
Total Alkalinity(mg/l)	320.2 (40.3)	336.1 (64.5)	350.9 (62.5)	412 (27.8)	<0.0001
Chloride(mg/l)	154.7 (16.4)	204.4 (11.1)	188 (18.8)	213.1 (21.6)	<0.0001
Total Hardness(mg/l)	342.2 (32.6)	379.9 (18.7)	342.6 (23.4)	390.1 (28.5)	<0.0001
Calcium Hardness(mg/l)	184.5 (25.5)	198.9 (29.5)	203.4 (29.4)	214.5 (30.1)	<0.0001
Magnesium Hardness(mg/l)	157.8 (28.6)	181 (14.5)	139.2 (23.3)	175.6 (17.8)	<0.0001
Nitrate Nitrogen(mg/l)	4.6 (0.8)	6.3 (3.2)	9.4 (2.6)	4.5 (0.7)	<0.0001
Total Phosphorus(mg/l)	3.4 (0.6)	3.1 (1.2)	7.1 (1.1)	3.2 (0.7)	<0.0001
Ortho Phosphorus(mg/l)	2.8 (0.8)	2.5 (1.1)	6.2 (1.1)	2.7 (0.7)	<0.0001
Organic Phosphorus(mg/l)	0.6 (0.2)	0.6 (0.4)	0.9 (0.5)	0.5 (0.3)	<0.0001
Free Co2(mg/l)	4.5 (3.9)	2.5 (6.1)	2.6 (4.3)	3.3 (3.1)	0.02
Total Coliform CFU/100 ml	60926.9 (69266)	16087.9 (15518.4)	44037.1 (32674.9)	89221.4 (138041.4)	<0.0001
Total E coli CFU/100 ml	4470.5 (6718.1)	214.3 (285.3)	3067.1 (3718.7)	1051.9 (1367.9)	<0.0001

Note: p-values are extracted from ANOVA; -: Not done.

Table S2. Water Quality Criteria.

Designated-Best-Use	Class of Water	Criteria
Drinking Water Source without conventional treatment but after disinfection	A	Total Coliforms Organism MPN/100ml shall be 50 or less<br>pH between 6.5 and 8.5<br>Dissolved Oxygen 6mg/l or more<br>Biochemical Oxygen Demand 5 days 20C 2mg/l or less
Outdoor bathing (Organised)	B	Total Coliforms Organism MPN/100ml shall be 500 or less pH between 6.5 and 8.5 Dissolved Oxygen 5mg/l or more<br>Biochemical Oxygen Demand 5 days 20C 3mg/l or less
Drinking water source after conventional treatment and disinfection	C	Total Coliforms Organism MPN/100ml shall be 5000 or less pH between 6 to 9 Dissolved Oxygen 4mg/l or more<br>Biochemical Oxygen Demand 5 days 20C 3mg/l or less
Propagation of Wild life and Fisheries	D	pH between 6.5 to 8.5 Dissolved Oxygen 4mg/l or more<br>Free Ammonia (as N) 1.2 mg/l or less
Irrigation, Industrial Cooling, Controlled Waste disposal	E	pH betwwn 6.0 to 8.5<br>Electrical Conductivity at 25C micro mhos/cm Max.2250<br>Sodium absorption Ratio Max. 26<br>Boron Max. 2mg/l
	Below-E	Not Meeting A, B, C, D & E Criteria

Source: http://cpcb.nic.in/water-quality-criteria/ (Accessed on April 30, 2018)- Central Pollution Control Board Ministry of Forest, Environment and Climate Change, Government of India.
